# Neurocutaneous features of tuberous sclerosis complex: A case report from Bangladesh

**DOI:** 10.1097/MD.0000000000042534

**Published:** 2025-05-16

**Authors:** Md Iftekher Iqbal, Fariah Osman

**Affiliations:** a Department of Glaucoma, Ispahani Islamia Eye Institute and Hospital, Dhaka, Bangladesh; b Department of Ophthalmology, Ispahani Islamia Eye Institute and Hospital, Dhaka, Bangladesh.

**Keywords:** adenoma sebaceum, cortical tubers, epilepsy, retinal astrocytic hamartoma, tuberous sclerosis complex.

## Abstract

**Rationale:**

A rare autosomal dominant disorder, tuberous sclerosis complex (TSC), presents with various symptoms from the beginning. It is characterized by neurological signs such as epilepsy, skin abnormalities, and the development of benign lesions in several organs. This study reviews the literature on different clinical and imaging presentations of TSC and treatment options. Additionally, we report a case in which a young boy presented to an ophthalmologist for an eye checkup and was later found to have TSC. This report aims to raise awareness among clinicians regarding such clinical scenarios.

**Patient concerns:**

A 9-year-old boy presented to an ophthalmologist for blurry, distant vision in both eyes.

**Diagnosis:**

Based on clinical features and neuroimaging, a diagnosis of TSC was established according to the 2012 International TSC Consensus with 6 primary features but without any secondary features.

**Interventions:**

The patient was prescribed haloperidol and intravenous diazepam as part of seizure management.

**Outcomes:**

The patient was referred to the neurology department. Initially, his seizure was controlled with anti-seizure medications. But as the patient was lost to follow-up, the long-term effect of anti-seizure medication on seizure control could not be evaluated.

**Lessons:**

This case report aims to enhance comprehension of the clinical diagnosis to prevent incorrect diagnosis, overlooked diagnosis, and suboptimal treatment.

## 1. Introduction

An autosomal dominant genetic disorder, tuberous sclerosis complex (TSC), is distinguished by the presence of numerous organ lesions that are closely associated with the TSC gene.^[1,2]^ Intractable epilepsy, facial angiofibroma, and mental impairment are its prominent features.^[3]^ It occurs around 1 in every 6000 to 10,000 people, and even though women typically experience less severe symptoms, the frequency of TSC is not different based on race or gender.^[4]^

A significant neurologic symptom of TSC patients is epilepsy, and between 62% and 93% of them are reported to have epilepsy.^[5,6]^ TSC-related epilepsies typically manifest in infancy and early childhood, but they may affect anyone at any age. Refractory epilepsy affects about half of the patients.^[7]^

The diagnosis can be established in the presence of common manifestations such as renal angiomyolipoma, white matter (WM) abnormalities, retinal abnormalities, cardiac rhabdomyoma, lymphangioleiomyomatosis, and cortical tubers or subependymal nodules (SENs) and can be confirmed by gene analysis. This is particularly significant when skin lesions are present.^[8]^

Imaging plays a crucial role in establishing the complete extent of involvement and in making an assumption-based diagnosis in TSC evaluations. Patients with TSC show various aberrant signals of extended longitudinal relaxation times (T1) and transverse relaxation times (T2) on magnetic resonance imaging (MRI), as well as subependymal “candle-drip”-shaped calcified nodules on brain computed tomography.^[8]^

As a result of the variable initial symptoms of TSC, patients with TSC are initially diagnosed in various departments. Underdiagnosis and misdiagnosis of this disease are prevalent as a consequence.^[3]^

It is imperative to undertake a multidisciplinary approach that includes regular monitoring from childhood to adulthood in order to effectively manage TSC. As a result of its multisystem involvement and varying severity, it is imperative to implement a comprehensive management strategy in order to reduce patient morbidity and mortality. Furthermore, when TSC is suspected, genetic counseling should be implemented.^[9]^

With the goal of improving knowledge of TSC’s typical features, this study reports on a case of TSC that was misdiagnosed as epilepsy and aims to minimize misdiagnosis and overtreatment of TSC. The TSC’s neuroimaging symptoms and guidelines for diagnosis are also reviewed.

## 2. Case presentation

A Bangladeshi 9-year-old boy visited an ophthalmologist with his father after a few months of blurry, distant vision in both eyes (BE).

According to the patient’s father, the patient was a child of non-consanguineous parents who used to watch television from a close distance and was noticed for almost 3 months. He also added that he had a history of convulsions several times at an unknown frequency since the age of 4. However, he was on irregular follow-ups with his primary physician with no documented previous medical history. His pre and postnatal periods were unremarkable, with normal vaginal delivery at term and immunization as per the country’s Expanded Program on Immunization schedules. He does not have a developmental delay, but his speech was not connected or relevant, which is a sign of an intellectual handicap; thus, he was unable to attend school. His speech was limited to short sentences. He was the only child of his parents, and none of the parents’ family members suffered from any neurological or skin disorders.

**Figure 1.**
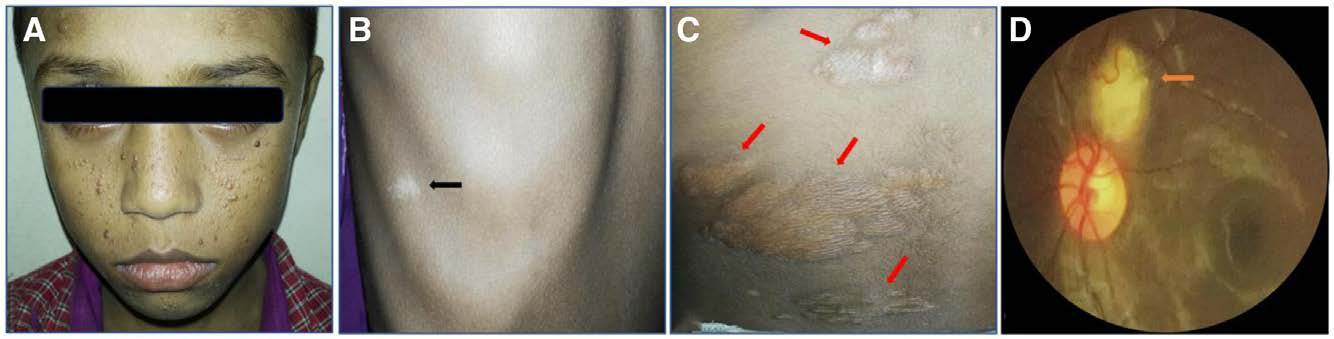
Presenting features of Tuberous Sclerosis Complex (TSC): (A) Adenoma sebaceum on face and forehead; (B) Ash leaf spot (black arrow); (C) Shagreen patches (multiple red arrows) on the back; (D) retinal astrocytic hamartoma (orange arrow) in left eye.

On general examination, the boy looked anxious and irritable, and there were facial angiomas (also known as adenoma sebaceum) all over his face and forehead (Fig. 1A). Further examination revealed an Ash leaf spot over the right hypochondriac region (Fig. 1B) and multiple Shagreen patches on the back (Fig. 1C).

The ophthalmic evaluation revealed his best-corrected visual acuity of 6/6 (−0.50 DSph) by the Snellen chart and intraocular pressure of 14 mm Hg by noncontact tonometer with no noticeable anterior segment abnormalities in BE. On the other hand, the dilated fundus exam showed a retinal astrocytic hamartoma about 2 mm × 2.5 mm in size, above the optic disc in the left eye (Fig. 1D).

Neurological evaluation revealed normal upper and lower limb tone, brisk deep tendon and superficial reflexes, and a bilateral negative Babinski sign. Other systems revealed no abnormalities. Other systems revealed no abnormalities.

Laboratory investigations, including complete blood count, serum electrolytes, and renal and liver function tests, were within normal range, and there were no detectable abnormalities in this patient’s echocardiography and abdominal ultrasonogram. However, we could not do genetic testing due to our country’s unavailability.

**Figure 2.**
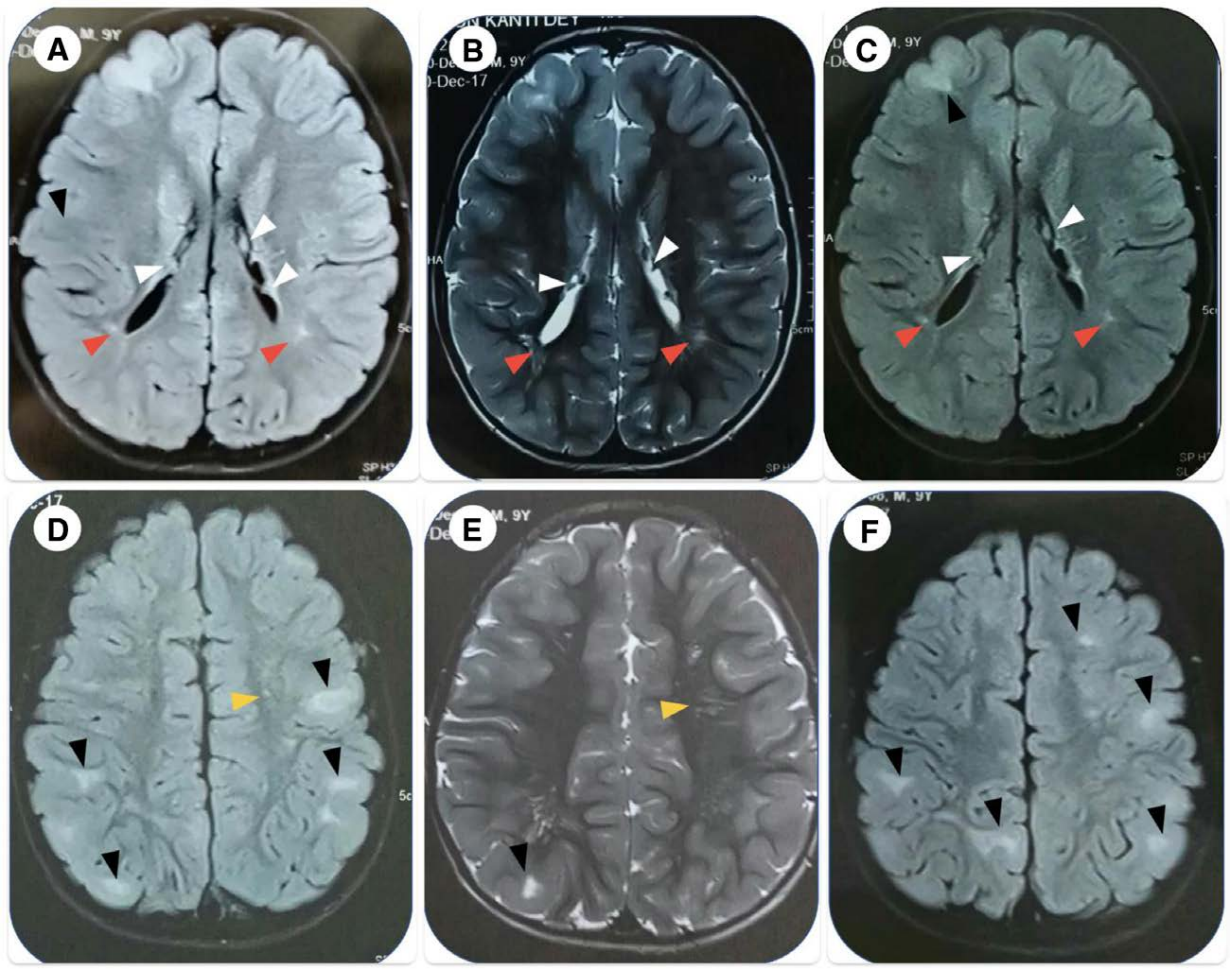
Axial MRI of the brain: cortical tubers (black arrowheads), subependymal nodules (white arrowheads), white matter lesions (red arrowheads), radial linear white matter lesions (yellow arrowheads). MRI = magnetic resonance imaging.

Axial MRI of the brain showed multiple, well-circumscribed lesions (black arrowheads) in the cortex: hypointense on T1-weighted images (Fig. 2A, D), hyperintense on T2-weighted images (Fig. 2B, E), and fluid-attenuated inversion-recovery (FLAIR) images (Fig. 2C, F), consistent with cortical tubers. There were multiple SENs along the ventricular surfaces of both lateral ventricles (white arrowheads): hyperintense on T1-weighted images (Fig. 2A) and iso-to-hyper intensity on T2-weighted (Fig. 2B) and FLAIR images (Fig. 2C). FLAIR images (Fig. 2C) demonstrated small, cyst-like, well-demarcated, hyper-intense lesions near the lateral ventricles (red arrowheads); these were hypo-intense on T1-weighted image (Fig. 2A) and hyper-intense on T2-weighted image (Fig. 2B), and suggestive of WM lesions (WMLs). There were thin, straight bands of radial linear WMLs (yellow arrowheads) also present as hyper-intense lesions on the T2-weighted image (Fig. 2E) and iso-to-hypointense lesions on the T1-weighted image (Fig. 2D).

Finally, the patient was diagnosed with a case of TSC based on his history and comprehensive clinical and MRI examinations with the following 6 primary features: facial angiofibroma, hypopigmented macules (Ash leaf spot), Shagreen patch, retinal astrocytic hamartoma, cortical dysplasia (cortical nodules or WM radial migration lines), and SENs. He did not have any secondary features.

After proper counseling, we referred him to the neurology department, where they prescribed haloperidol (1 mg b.i.d.) and, if a convulsion happened, intravenous diazepam (1 mg) slowly and with regular follow-up. Unfortunately, this patient was lost to follow-up.

## 3. Discussion

Defects in the TSC1/TSC2 complex, an inhibitor of the mammalian target of rapamycin pathway, result in TSC, a congenital condition.^[10]^ The majority of individuals (around 80%) are diagnosed during childhood, although in rare cases, the usual neurological symptoms and skin characteristics may not present until late childhood or adulthood, delaying the diagnosis.^[11]^ In addition to improving treatment outcomes and reducing needless medical expenditures, early detection can improve the prognosis for people with TSC. We report this case of a 9-year-old boy who was initially misdiagnosed with epilepsy and came for an ocular evaluation for blurry, distant vision in BE. This case should help increase understanding of TSC.

Epilepsy is the most common neurological manifestation in TSC patients.^[5]^ Nearly all TSC patients will develop epilepsy after their first seizure event. Therefore, doctors must keep a close eye on the patient. While TSC-related epilepsy typically manifests in the first few months of birth, it can also manifest later in life. It is most frequent in children. Seizure patterns might change over time; however, focal seizures are the most prevalent.^[12]^ Our patient also had a history of occasional seizures from early childhood and was underdiagnosed as a case of epilepsy only.

Clinical characteristics are the primary indicators for a TSC diagnosis at the initial phase. The traditional, or Vogt triad of facial angiofibroma, epilepsy, and mental retardation is not the full range of presentation associated with TSC, as discovered by genetics and medical imaging. Table 1 shows the updated TSC diagnostic criteria that were published in 2012. These criteria include the following items: First, a diagnosis of TSC can be confirmed by pathogenic mutations in the TSC1 or TSC2 gene; second, in order to prove a clinical diagnosis of TSC, it is necessary to have 2 main features or 1 main feature and 2 secondary features at the same time; and third, it is possible to suspect that 1 main feature or 2 secondary features indicate TSC.^[13]^

**Table 1:** Consensus statement of the 2012 International Tuberous Sclerosis Complex (TSC) Consensus Conference.

Genetic testing	Clinical diagnostic criteria	
	Primary features	Secondary features
Definite loss-of-function mutations in the TSC1 and/or TSC2 genes with dysregulation of the mTOR pathway (which is sufficient but not necessary for the diagnosis of TSC). TSC1 and/or TSC2 mutations that are unclear or of no functional significance do not meet these criteria, and therefore, they cannot be used in the diagnosis of TSC.	Facial angiofibromas (≥ 3) or frontal fibrous plaque	Multiple dental enamel pits (≥ 3)
	Hypopigmented macules (≥ 3 with a minimum diameter of 5 mm)	Oral fibromas (≥ 2)
	Ungual fibromas (≥ 2)	Nonrenal angiomyolipoma
	Shagreen patch	Multiple renal cysts
	Multiple retinal nodular hamartomas	Retinal achromic patch
	Cortical dysplasia (cortical nodules or white matter radial migration lines)	“Confetti” skin lesions
	Subependymal nodules	
	Subependymal giant cell astrocytoma	
	Cardiac rhabdomyoma	
	Pulmonary lymphangioleiomyomatosis	
	Renal angiomyolipomas (≥ 2)	

mTOR = mammalian target of rapamycin.

The importance of imaging in diagnosing TSC was illustrated in a case report that included solitary subependymal giant cell astrocytoma as the initial imaging manifestation.^[14]^ Cortical tubers, WMLs, SENs, and subependymal giant cell astrocytoma are the 4 primary neuroimaging characteristics of TSC.^[3]^ Glial brain hamartomas, or cortical tubers, can affect both gray and WM. Overexpression of microRNA-34a in cortical cells may be linked to the development of these tumors. Although it can affect the entire brain, cortical tubers are primarily found in the frontal and parietal lobes.^[3]^

Hypopigmented macules on the right hypochondriac area, numerous Shagreen patches across the back of the patient’s torso, and symmetrical, reddish, hard, and waxy papules on both cheeks and the forehead were all present in the case we documented. His left eye also had a retinal astrocytic hamartoma. A cranial MRI of this patient showed cortical tubers, SENs, and WMLs. As a result, the documented case satisfied the clinical diagnostic requirements listed in Table 1.

Seizure control and TSC lack a conclusive treatment. The first-line treatment for focal seizures before the age of 1 year is GABAergic medication (e.g., vigabatrin). As an antiepileptic medication, levetiracetam can lessen the frequency of seizures in TSC patients. Medications used for symptomatic relief include sodium valproate, phenobarbitone, carbamazepine, benzodiazepine, oxcarbazepine, topiramate, and oral steroids. When treating TSC patients, multiple medications are administered if required.^[12]^ There are also studies regarding the ketogenic diet, surgery (temporal lobectomy, corpus callosotomy), laser or thermal ablation of the seizure-causing area of the brain, and vagus nerve stimulation in TSC with epilepsy management.^[9]^ In our case, we referred him to the neurology department for further evaluation and management, where they prescribed benzodiazepines: haloperidol (1 mg b.i.d.) routinely and slow intravenous diazepam (1 mg) for acute seizure control. However, the patient was advised to follow up regularly but eventually lost track of follow-up. Here, the patient’s financial situation and the availability of the medications determined how they were utilized.

The limitation of this case report is the unavailability of genetic testing facilities in the country; chest computed tomography was not conducted due to resource constraints and the lack of long-term follow-up.

## 4. Conclusion

Early detection of distinctive dermatological lesions, which may manifest at any point throughout childhood, is crucial for diagnosing TSC. Additionally, neuroimaging is vital for this condition’s early diagnosis, monitoring, and therapy. Given that the majority of individuals with TSC have epilepsy, it is crucial to promptly manage and avoid seizures following diagnosis. Doing so may enhance cognitive neurodevelopment and positively influence the overall quality of life.

## Acknowledgments

The Authors thank Ophthalmology and Neurology Department of Chittagong Medical College, Chittagong – 4203, Bangladesh.

## Author contributions

**Conceptualization:** Md Iftekher Iqbal.

**Data curation:** Md Iftekher Iqbal, Fariah Osman.

**Formal analysis:** Md Iftekher Iqbal, Fariah Osman.

**Methodology:** Md Iftekher Iqbal.

**Supervision:** Md Iftekher Iqbal.

**Visualization:** Md Iftekher Iqbal.

**Writing – original draft:** Md Iftekher Iqbal, Fariah Osman.

**Writing – review & editing:** Md Iftekher Iqbal.
